# Sensorimotor expertise effects extend from dance to face perception

**DOI:** 10.1093/cercor/bhag186

**Published:** 2026-11-07

**Authors:** Vasiliki Meletaki, Bettina Forster, Beatriz Calvo-Merino

**Affiliations:** Centre for Cognition and Neuroscience, Department of Psychology and Neuroscience, City St George’s, University of London, Northampton Square, London, EC1V 0HB, United Kingdom; Penn Center for Neuroaesthetics, University of Pennsylvania, 3 West Gates Building, 3400 Spruce St, Philadelphia, PA, United States; Centre for Cognition and Neuroscience, Department of Psychology and Neuroscience, City St George’s, University of London, Northampton Square, London, EC1V 0HB, United Kingdom; Centre for Cognition and Neuroscience, Department of Psychology and Neuroscience, City St George’s, University of London, Northampton Square, London, EC1V 0HB, United Kingdom

**Keywords:** dance, expertise, embodiment, emotion, face, somatosensory

## Abstract

Domain specific expertise affects neural and behavioral processes of perception and memory. We explore if sensorimotor expertise extends to other relevant social mechanisms, such as the neural processing of emotional faces. We recorded brain activity of 26 dancers and 26 non-dancers using visual and somatosensory evoked potentials (VEPs, SEPs) during an emotion and sex discrimination task on happy, afraid, and neutral faces. We predicted expertise-related modulation of visual facial emotion processing in visual and somatosensory areas. EEG results revealed distinct visual and somatosensory ERP patterns in the two groups. VEP findings showed that dancers distinguished between neutral and emotional faces at N170 and P200. Non-dancers differentiated between the three emotions with more lateralized processing. For SEPs, dancers uniquely showed early emotion effects (P50) in central somatosensory regions and task-modulated somatosensory engagement at later stages (N80, P100). Non-dancers showed later, localized somatosensory effects. In summary, dance expertise shapes both visual and somatosensory processing of facial emotion, indicating a broader neural representation of emotions. These findings provide novel evidence for a differential facial emotion processing in dancers, extending the dance expertise modulation beyond perception of dance-specific stimuli, to face emotion perception. Overall, this work highlights the strong influence of sensorimotor training on emotion perception.

## Introduction

Artistic expression, especially in the performing arts, offers a powerful route for communicating and sharing emotion. Professional dancers develop refined embodied awareness through prolonged, precise sensorimotor training, a quality often invoked to explain why dance-based approaches are effective in clinical and rehabilitative contexts (Kiepe et al. 2012; Koch et al. 2014; Meekums et al. 2015; Fancourt and Finn 2019). The impact of sensorimotor training in emotion, perception and cognition has been classically described in studies focusing on behavioral, physiological, and neural processes (Bläsing et al. 2012; Karpati et al. 2015; Christensen et al. 2017a; Fink et al. 2021). However, this research has focused nearly exclusively on processing movement or body-related dance information (Christensen and Calvo-Merino 2013). Here, we aim to go beyond the well-known dance domain-specific expertise effect, and we ask whether dancer’s sensorimotor training also modulates other common perceptual processes. In particular, given the importance of the embodied framework in perception of emotions, we ask if dance training modulates how the brain processes emotional information in other social contexts, specifically the perception of facial expressions.

From a purely movement observation perspective, neuroimaging studies with expert dancers have shown increased brain activity in the action observation network when viewing familiar dance movements, especially repertoire-specific (Calvo-Merino et al. 2005; Calvo-Merino et al. 2006; Cross et al. 2006). Electrophysiological studies further indicate differentiated neural processing in dancers during dance movement observation, including delayed alpha/beta desynchronization (Orgs et al. 2008), modulation of ERP components like N400 and Late Positivity (Orlandi et al. 2017), and enhanced early occipito-temporal cortex engagement (Orlandi and Proverbio 2019). Structural and functional adaptations due to sensorimotor art training have also been explored. For example, dancers showed decreased gray/white matter volumes in motor cortex that scale with training onset (Hänggi et al. 2010), and differential white matter diffusivity was described between dancers and musicians (Giacosa et al. 2016).

Enhanced emotional sensitivity and empathy have been reported across performing art disciplines (Goldstein and Bloom 2011; Jola et al 2021;  Sevdalis and Keller 2011; Sevdalis and Raab 2014; Schirmer-Mokwa et al. 2015; Schmidt et al. 2021; Sokol-Hessner et al. 2022). For dancers, years of practice cultivate fine motor control and spatial precision and have been linked to interoceptive, proprioceptive and body awareness (Jola et al. 2011; Christensen et al. 2017b; Rein et al. 2011). Perception of affective familiar dance body movement is also enhanced through dance, as shown in physiological studies using galvanic skin response (Christensen et al. 2016a), facial electromyography (Kirsch et al. 2016) and in behavior (Kirsch et al. 2013; Kirsch et al. 2015;  Christensen et al. 2016b). Whether these enhanced emotion effects are associated to a more defined visual sensitivity in dancers (Calvo-Merino et al. 2010) or to a general enhanced empathy (Izountouemoi and Esteves 2023) requires further research. Interestingly, interoceptive accuracy has been associated to emotional empathy and sensitivity to others' emotional states (Herbert and Pollatos 2012), suggesting that dancers' enhanced body awareness may be at the heart of how they process emotional signals from others.

Embodied simulation accounts propose that perceiving others' emotions recruits sensorimotor representations of those emotional states in the observer (Niedenthal 2007; Niedenthal and Maringer 2009;  Keysers et al. 2010; Wood et al. 2016). If dance training refines these sensorimotor-emotional representations, then the resulting neural differences should be observable not only for dance-related stimuli but for other emotional signals too, such as facial expressions. Whether this increased emotion sensitivity is domain-specific (tied to practiced bodily expressions) or generalizes to other formats such as everyday facial expressions remains unresolved. Related work in visual arts suggests that artistic expertise can tune face processing (portrait perception in painters by Seabra et al. 2022), motivating the present test in dancers.

Facial emotion perception engages occipital and temporal cortices (Adolphs 2002), with early occipital stages (e.g. P120, N170, and P200) extracting visual features and later temporal stages supporting recognition and meaning (Haxby et al. 2000; Vuilleumier and Pourtois 2007; Calvo and Nummenmaa 2016; Liu et al. 2021). Embodied accounts argue that somatosensory cortices directly contribute to perceiving others’ emotions, in addition to visual regions (Sel et al. 2014, 2020; Arslanova et al. 2023), with causal evidence for early somatosensory involvement in facial emotion processing (Pourtois et al. 2004; Pitcher et al. 2008). Interestingly, this visual and early somatosensory embodied processes associated to face emotion perception have shown different patterns in neurodevelopmental disorders, such as individual with autism spectrum disorder compared to typically developed individuals (Fanghella et al. 2022, 2026).

Here we bridge these literatures by asking whether professional ballet dancers show distinctive *visual* and *somatosensory* processing of facial emotions. We recorded visual evoked potentials (VEPs) and somatosensory evoked potentials (SEPs) while dancers and non-dancers performed an emotion-discrimination task on faces, with a sex-discrimination task as a control task. To probe somatosensory processing, we delivered brief tactile stimulation to the left index finger on half of the trials. Accordingly, we expected strongest SEP differences contralateral in right somatosensory cortex. To explore somatosensory effects beyond visual carry over effects, we employed a SEP/VEPs subtraction method following similar studies (Sel et al. 2014; Fanghella et al. 2022). Hence, we subtracted the activity from visual trials from the visual and tactile trials to keep only the somatosensory processing of the facial emotion effects (see Fig. 1).

**Figure 1:**
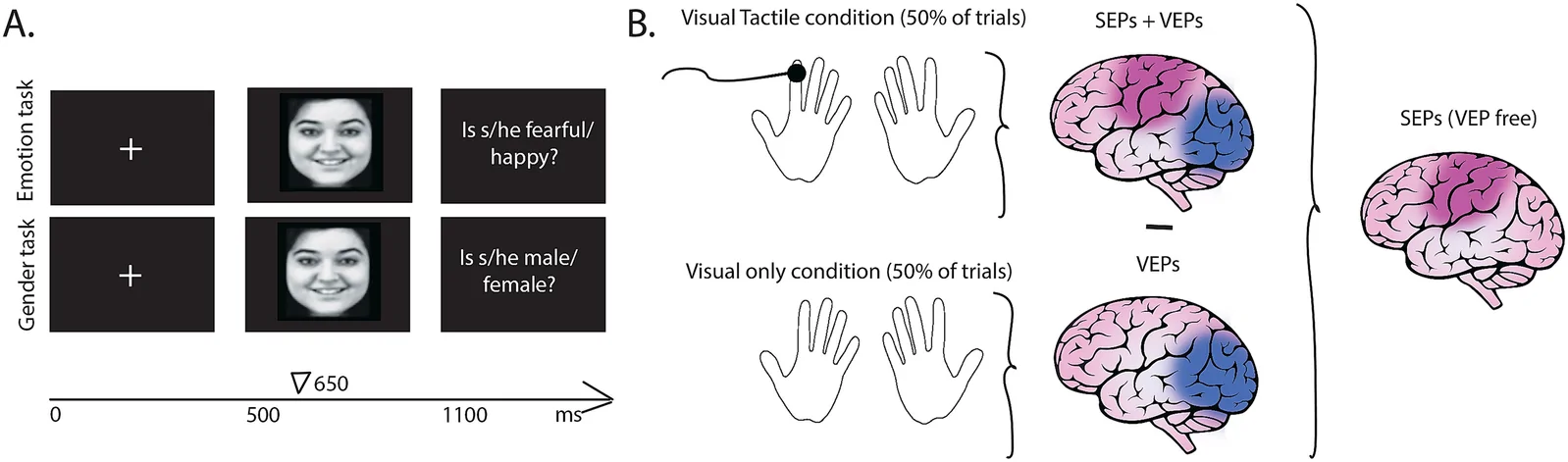
Procedure and VEP/SEP subtraction method. *Note*: A. Trial example and task: face stimuli were presented at 500 ms from fixation cross onset and on 50% of trials tactile stimulation was delivered on the left index finger at 105 ms after the face onset (605 ms after fixation cross onset). On 10% of trials, a question appeared after 1100 ms. For emotion discrimination task: “Is s/he fearful?” or “Is s/he happy?”. For sex discrimination task: “Is s/he male?” or “Is s/he female?”. B. Subtraction of visual-only condition, with no tactile stimulation, from visual-tactile condition, when tactile stimulation was delivered. This method allowed us to isolate somatosensory evoked activity from visual carry-over effects (VEP + SEP) – (VEP) = SEP (VEP free) (Galvez-Pol et al. 2020a; Fanghella et al. 2022, 2026; Sel et al. 2014).

Based on the above evidence showing that dance expertise shapes perception of body and movement, as well as dance specific movement expressions, we hypothesize that dance expertise will also influence facial emotion perception. We predicted that dancers would show different ERP responses to facial emotions than the non-dancers, reflecting modulation in both visual (P120, N170, and P200) and somatosensory stages (P50, N80, P100, and N140). We did not specify directional or topographic predictions for VEPs or SEPs beyond the contralateral SEP expectation. This design directly tests whether dancers’ emotion sensitivity generalizes from body actions to faces, thereby clarifying how artistic and sensorimotor expertise shape core mechanisms of human emotion perception.

## Materials and methods

### Participants

Twenty-six professional or trainee ballet dancers (22 females, 1 left-handed, M_age_ = 26.61 years, SD = 5.3, range 19 to 38 years, M_dance experience_ = 17.38 years, SD = 6.8, M_professional experience_ = 4.86 years, SD = 4, M_ballet training_ = 7 h/week, SD = 5.4, M_other dance styles training_ = 9.97 h/week, SD = 6.15) and twenty-six control participants with no dance experience (22 females, 2 left-handed, M_age_ = 25.59 years, SD = 4.4, range 21 to 35 years) participated in the study. The dancers were recruited from different dance schools in London (Pineapple Dance Studios, Rambert Dance and more). The control participants were recruited via an online recruitment platform (SONA Inc.) and matched to dancers for age and sex. Control participants were screened for artistic and sports expertise during recruitment and none reported significant experience ensuring that they did not have substantial sensorimotor or artistic training. All participants reported normal or corrected-to-normal vision, were naive to the study's objective, and provided written informed consent prior to their participation. The study was approved by the Psychology Ethics Committee of City St George’s, University of London (ETH1819-1868, approved on 2018 July 1). Participants received £8/hour for reimbursement.

### Stimuli

60 grayscale images of faces (half male) displaying happy, afraid, and neutral expressions from the Karolinska Directed Emotional Faces set (KDEF) (Lundqvist et al. 1998) were presented within a 1.4 × 1.57 inch rectangular frame. The images were used in a previous study where the happy and afraid expressions were rated high in valence, and neutral expressions low in valence (Sel et al. 2014).

### Psychometric measures

In line with our previous research (Christensen et al. 2017b; Arslanova et al. 2023; Fanghella et al. 2022), to assess group differences in relevant personality traits, participants completed the Beck Depression Inventory II (BDI-II) (Beck et al. 1987), the Twenty Item Toronto Alexithymia Scale-II (TAS-20) (Bagby et al. 1994), the State–Trait Anxiety Inventory for Adults (STAI) (Spielberger et al. 1983), the Multidimensional Assessment of Interoceptive Awareness (MAIA) (Mehling et al. 2012), and the 20-Item Prosopagnosia Index (PI-20) (Shah et al. 2015). Descriptive statistics for psychometric measures are presented in Table 1.

**Table 1:** Mean, SD, minimum and maximum scores on the questionnaires (TAS, STAI- state, STAI- trait, BDI, MAIA and the 20-item prosopagnosia index) for dancers and control participants.

Dancers	TAS	STAI- state	STAI- trait	BDI	MAIA	PI-20
Mean	41.04/37.5	36.81	42.04	8/6	3.69***	36.19
SD	12.18/51	7.99	10.31	6.08/18	.48	7.61
Range	27-82	21-51	25-65	1-19	2.68-4.75	25-56
**Non-dancers**	**TAS**	**STAI- state**	**STAI- trait**	**BDI**	**MAIA**	**PI-20**
Mean	45.68	36.32	42.29	5.96	2.71***	37.12
SD	12.01	11.11	12.36	5.87	.66	10.96
Range	21-72	20-56	21-73	0-23	1.49-3.82	22-70

Note: TAS: Toronto Alexithymia Scale, STAI-state: State Trait Anxiety Inventory- State scale, STAI-trait: State Trait Anxiety Inventory- Trait scale, BDI: Beck Depression Inventory, MAIA: Multidimensional Assessment of Interoceptive Awareness. *n* = 24 for STAI – trait anxiety for non-dancers because the data for one control participant was not recorded. The second values shown on TAS and BDI for dancers correspond to the median and range respectively due to normality tests showing non normal distribution, *D* (26) = 0.18, *P* = 0.027 for TAS and *D* (26) = 0.18, *P =* 0.029 for BDI. ***: the one-way ANOVA between groups showed that dancers have significantly higher mean MAIA score than non-dancers at *P* < 0.001.

### Procedure

Participants were seated in a dimly lit, sound-attenuated, electromagnetically shielded room, 80 cm from a computer monitor. Tactile stimulation was delivered via a 12 V solenoid with a blunt conical tip, applied to the tip of the left index finger. White noise, presented through a loudspeaker 90 cm from the participant, masked solenoid noise. Participants were instructed to ignore tactile stimuli and white noise.

Each trial began with a 500 ms fixation cross, followed by a 600 ms presentation of a neutral, happy, or afraid face (see Fig. 1A). In visual-tactile conditions, a tactile stimulus was delivered 105 ms after visual stimulus onset (Pitcher et al. 2008; Sel et al. 2014; Galvez-Pol et al. 2020a, 2020b; Fanghella et al. 2022). Visual-only conditions (50% of trials) presented visual stimuli without tactile stimulation to control for visual carry-over effects (Arslanova et al. 2019, Galvez-Pol et al. 2020a, 2020b). To set the different conditions (emotion or sex discrimination task) and to ensure sustained attention to the task throughout the EEG recording, in 20% of the trials per condition the images were followed by the question “Is s/he fearful?” or “Is s/he happy?” for the emotion discrimination task or “Is s/he male?” or “Is s/he female?” for the control sex discrimination task (see Fig. 1B). Each task comprised 600 trials in three blocks with breaks. Verbal yes/no responses were collected to avoid motor artifacts. Prior to testing, participants completed a practice session. Stimuli were presented centrally on a black background using E-prime 2.0 software (Psychology Software Tools) on a Windows 7 computer.

Following EEG tasks, participants completed subjective ratings of emotion and sex using visual analogue scales (VASs) with anchors “happy”- “neutral”- “afraid” and “male”- “female,” respectively. Intensity of VAS ratings was measured. Psychometric questionnaires were administered online via Qualtrics. Task order was counterbalanced across participants.

### E‌EG recording and data analysis

Continuous EEG was recorded using 64 active electrodes (EasyCap M10) mounted equidistantly on an elastic cap, referenced online to the right earlobe and offline to the average of all electrodes. Vertical and horizontal electrooculograms (EOG) and electrocardiogram (ECG) were recorded. EEG data were acquired using a BrainAmp amplifier (500 Hz sampling rate) and BrainVision Recorder. Offline analysis was conducted in BrainVision Analyzer 2.1. Noisy or flat-lined electrodes were spline interpolated. Data were filtered at 0.1-30 Hz (Butterworth zero-phase shift), re-referenced to average, and segmented into 700 ms epochs (−100 ms to 600 ms relative to tactile onset for SEPs, or visual onset for VEPs), baseline-corrected to the -100 ms to 0 ms pre-stimulus interval, and ocular artifact corrected (Gratton et al. 1983). Artifact rejection was applied to trials with amplitudes exceeding ±100 μV.

Single-subject ERPs were averaged for each condition (visual-tactile, visual-only), emotion (neutral, afraid, and happy), and task (emotion, sex discrimination). VEP-free SEPs were derived by subtracting visual-only ERPs from visual-tactile ERPs at the single-subject level (Galvez-Pol et al. 2018, 2020a, 2020b; Fanghella et al. 2022 and 2026; Sel et al. 2014), see Fig. 1B. Grand averages were computed for dancers and non-dancers for each emotion. Mean amplitudes were extracted for VEP components P120 (110 to 150 ms), N170 (156 to 196 ms), and P200 (222 to 262 ms) at occipital electrodes 42, 44, 54, 55, 57, and 58 (corresponding to O1/2, O9/10, PO9/10 of the 10-20 system). SEP components P50 (38 to 68 ms), N80 (70 to 98 ms), P100 (100 to 118 ms), and N140 (120 to 144 ms) were analyzed at 18 somatosensory electrodes corresponding to Fc1/2, Fc3/4, Fc 5/6, C1/2, C3/4, C5/6, Cp1/2, Cp3/4, Cp5/6 following Fanghella et al. (2022) and Sel et al. (2014). Time windows for all components were determined by visually inspecting grand average waveforms to center each window on the peak (Luck and Gaspelin 2017; Fanghella et al. 2022).

Repeated-measures ANOVAs were conducted for each time window with within-subject factors of task (emotion, sex), emotion (neutral, afraid, and happy), hemisphere (left, right), and electrode for VEPs or region (anterior, central, and posterior) and site (dorsal, dorsolateral, and lateral) for SEPs, and between-subject factor of group (dancers, non-dancers). Greenhouse–Geisser corrections were applied when sphericity was violated, and Bonferroni corrections were used for post-hoc pairwise comparisons for significant effects. Following Sel et al. (2014) and Arslanova et al. (2023), when the main effect of emotion was significant, we calculated the “emotion index” by subtracting the mean amplitudes of neutral condition from the mean amplitudes of afraid and happy emotion conditions to isolate the emotion effects on visual and somatosensory activity. One outlier (>3 SD of SEP amplitude across time windows) was excluded from analyses.

### Event Related Potential (ERP) signal-to-noise ratio

To verify comparable signal-to-noise ratios across conditions, a repeated-measures ANOVA with factors task (emotion or sex discrimination), condition (visual-tactile/visual-only condition), and emotion (neutral, afraid, happy) was performed on the total number of trials for the SEPs from the somatosensory electrodes. No significant main effects or interactions were found, indicating comparable signal quality across conditions, *P* > .05. Follow up paired t-tests compared the visual-tactile and tactile-only conditions on the two tasks and confirmed no significant differences in the number of trials between conditions for either task, *P* > .05.

## Results

### Behavioral performance and psychometric measures

#### Facial emotion and sex discrimination question trials during EEG

The mean behavioral accuracy in verbal responses for the emotion and sex discrimination tasks of each participant was calculated with a value between 0% (no correct answers) and 100% (100% correct answers) from the 20% of the trials that included the discrimination question. Behavioral accuracy was high overall (≥95%). An ANOVA with task (emotion, sex discrimination) and group (dancers, non-dancers) revealed a significant main effect of task, F(1, 48) = 5.34, *P* = .025, η_p_^2^ = .10, with higher accuracy for sex (M = 0.96, SD = 0.04) than emotion discrimination (M = 0.95, SD = 0.04). The task × group interaction was also significant, F(1, 48) = 7.67, *P* = .008, η_p_^2^ = .14, showing that dancers outperformed non-dancers in the sex task (98% vs. 95%), while accuracy did not differ between groups in the emotion task (94% vs. 95%). The main effect of group was not significant, *P* = .488, η_p_^2^ = .01.

#### Subjective emotion and sex discrimination ratings

To ensure that participants were able to distinguish between the three emotions and the sex of the faces presented, we scored their responses on the separate emotion and sex discrimination task on a VAS. We ran an emotion (neutral, afraid, happy) x group (dancers, non-dancers) 3 × 2 ANOVA for the emotion task and a sex (male, female) x group 2 × 2 ANOVA for the sex task. On the emotion task we found a main effect of emotion, F (2, 98) = 1750.52, *P* < .001, η_p_^2^ = .973 with Bonferroni post hoc comparisons showing a difference between the three emotions (M = 11, SD = 7.1 for afraid, M = 50.6, SD = 2 for neutral and M = 91, SD = 6.9 for happy, all *P* < .001). No significant differences were found between groups (group effect F (1,49) = 0.335, *P* = .565 and emotion x group interaction F (2, 98) = 0.234, *P* = .792). The ANOVA on the sex discrimination task showed a main effect of sex, F (1, 49) = 4,634.33, *P* < .001, η_p_^2^ = .99 with Bonferroni post hoc comparisons showing a difference between ratings for male and female faces, (M = 4.87, SD = 5.5 for female and M = 95, SD = 0.4.9 for male, *P* < .001). No significant group effect (F(1,49) = 0.223, *P* = .639) or sex x group interaction (F (1, 49) = 0.964, *P* = .331) was found.

#### Questionnaires

One-way ANOVAs revealed significantly higher MAIA scores in dancers compared to non-dancers (F(1, 49) = 36.88, *P* < .001), particularly the average MAIA score and the subscales related to noticing, attention regulation, emotion awareness, self-regulation, body listening, and trusting the body. No significant group differences were found for other psychometric measures (BDI-II, TAS-20, STAI, PI-20) (Table 1).

### Group differences on Visual Evoked Potentials

Repeated measures ANOVAs on VEP mean amplitudes included within-subject factors of task (emotion, sex), hemisphere (left, right), electrode (42, 55, 54 on the right and 44, 57, 58 on the left hemisphere, corresponding to O1/2, O9/10 and PO9/10), and emotion (neutral, afraid, happy), and between-subject factor of group (dancers, non-dancers). The key finding for VEPs was a modulation of emotion processing by dance expertise, observed in interactions involving the group factor across P120, N170, and P200 components. Because of our interest in expertise effects, we followed up and report interactions primarily with group, emotion, and task.

#### P120 (110–150 ms): Early expertise effect in occipital activity

At the earliest VEP component, a significant group x electrode interaction was found, F(1.36, 66.54) = 7.72, *P* = .003, η_p_^2^ = .136. Follow-up analyses showed a main effect of group at electrode O1, F(1, 49) = 4.79, *P* = .033, η_p_^2^ = .089, with dancers and non-dancers differing in occipital activity, suggesting early modulation of visual processing by dance expertise.

#### N170 (155–195 ms): Expertise effect on emotion processing

Dance expertise modulated emotion processing at the N170 component. While both groups showed main effects of emotion, the pattern differed. Dancers showed reduced negativity for neutral faces compared to emotional faces (afraid, happy), whereas non-dancers showed a more graded and lateralized emotion effect with distinct responses to neutral, afraid and happy faces. Group differences were also observed for neutral faces specifically, with dancers showing greater negativity (Fig. 2).

**Figure 2:**
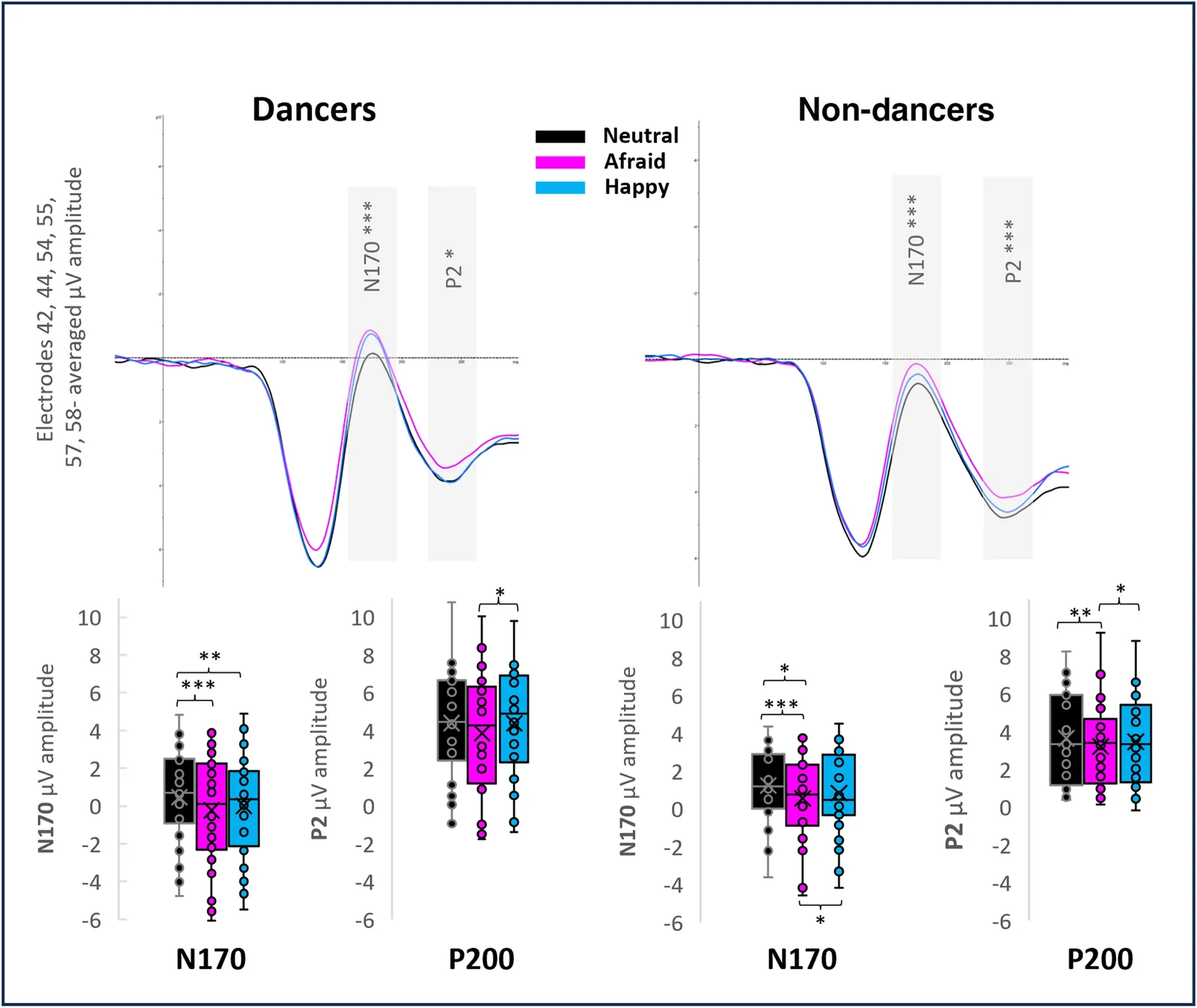
Averaged ERP activity over the six occipital electrodes 42, 44, 54, 55, 57, 58 corresponding to O1/2, O9/10 and PO9/10 for the emotion effect on each group. *Note*: The top two figures show the ERP waves averaged across the six electrodes for each neutral, afraid and happy faces for dancers and non-dancers. The boxplots below show the VEP amplitude distribution and individual data points for neutral, afraid and happy emotion condition at N170 and P2 for each group. At N170 dancers had a significant main effect of emotion with neutral eliciting less negativity than afraid and happy expression. Non-dancers had a significant main effect of emotion with neutral eliciting more positivity than afraid and happy, and afraid more positivity than happy. At P2 dancers had a significant emotion effect with afraid expression eliciting less positivity than happy. Non-dancers had a significant emotion effect with afraid expression showing less positivity than neutral and happy. VEP amplitude for neutral expressions is depicted in black, for afraid expressions in magenta and for happy expressions in blue. ^*^ for *P* < .05, ^**^ for *P* < .01 and ^***^ for *P* < .001.

The significant group x emotion x hemisphere x electrode interaction (F(3.144, 154.077) = 2.711, *P* = .044, η_p_^2^ = .052) was followed for each group (Fig. 2). For dancers, a main effect of emotion (F(2, 50) = 9.784, *P* < .001, η_p_^2^ = .281) indicated that neutral faces elicited less negative N170 amplitudes compared to both afraid (t (25) = 3.61, *P* = .004) and happy faces (t(25) = 3.36, *P* = .007) suggesting that dancers differentiated neutral from emotional expressions at this early stage. Non-dancers also exhibited a main effect of emotion (F(2, 48) = 16.858, *P* < .001, η_p_^2^ = .413) with a more graded emotion sensitivity: neutral faces elicited more positive amplitudes compared to afraid (t (24) =5.49, *P* < .001) and happy (t(24) = 2.77, *P* = .032), and afraid faces elicited more positive amplitudes than happy faces (t(24) = 3.32, *P* = .009). Furthermore, non-dancers showed an emotion x hemisphere x electrode interaction (F(4, 96) = 3.448, *P* = .011, η_p_^2^ = .126), with a more pronounced emotion effect in the right hemisphere (emotion x electrode interaction: F(4, 96) = 4.332, *P* = .003, η_p_^2^ = .153), suggesting a more lateralized emotion processing in non-dancers compared to the more global effect observed in dancers.

To examine where groups differed for specific emotion categories, we followed the group x emotion x hemisphere x electrode interaction also by each emotion (neutral, afraid, happy). The neutral condition revealed a group x electrode interaction (F(1.29, 63.011) = 4.100, *P* = .037, η_p_^2^ = .077). When averaging across homologous electrodes (since there was no significant hemisphere effect), a main effect of group emerged for the electrode pair O10/PO10 (F(1, 50) = 6.452, *P* = .014), with dancers showing more negative N170 amplitudes than non-dancers specifically for neutral faces.

#### P200 (222–262 ms): Expertise effect continues to later stage emotion processing

Similar to N170, dance expertise modulated emotion processing at the P200 component. The interaction pattern mirrored that of N170, with dancers showing differential processing of afraid vs happy faces, while non-dancers showed differentiation between afraid vs neutral/happy. Non-dancers also showed a more lateralized emotion effect at P200 (Fig. 2).

The significant group x emotion x hemisphere x electrode interaction observed at P200 (F(4, 196) = 3.197, *P* = .014, η_p_^2^ = .061) was followed for each group (Fig. 2). Dancers showed a main effect of emotion (F(2, 50) = 3.959, *P* = .025, η_p_^2^ = .137), with afraid faces eliciting less positive P200 amplitudes than happy faces (t(25) = 2.85, *P* = .026), and a more negative afraid-neutral emotion index compared to the happy-neutral index (t(25) = −2.842, *P* = .009). This suggests that dancers differentiated between negative (afraid) and positive (happy) emotions in P200. Dancers also showed an emotion x electrode interaction (F(4, 100) = 4.622, *P* = .002, η_p_^2^ = .156) that we explored by averaging homologous electrodes (since there was no significant hemisphere effect) and found main effects of emotion for electrode pairs O9 and PO9 (F(2,50) = 4.912, *P* = .011, η_p_^2^ = .164) and O10 and PO10 (F (2, 50) = 6.147, *P* = .004, η_p_^2^ = .1697). For both electrode pairs, afraid faces elicited less positive amplitudes than happy faces (t(25) = −3.02, *P* = .017 and t(25) = −3.85, *P* = .002), and the afraid-neutral emotion index was more negative than the happy-neutral index (t(25) = −3.03, *P* = .006 and t(25) = −3.86, *P* = .001).

Control participants showed a main effect of emotion (F(2, 48) = 9.924, *P* < .001, η_p_^2^ = .293), but with a different pattern than dancers: afraid faces elicited less positive amplitudes than both neutral (t(24) = −3.90, *P* = .002) and happy faces (t(24) = −3.14, *P* = .013), and the afraid-neutral emotion index was more negative (t(24) = −3.143, *P* = .004). Non-dancers also showed an emotion x hemisphere x electrode interaction (F(4, 96) = 2.988, *P* = .023, η_p_^2^ = .111), with main emotion effects in both hemispheres and emotion x electrode interactions in the right hemisphere, mirroring the lateralization observed at N170. Further analysis of the emotion x electrode interaction (F(4, 96) = 2.624, *P* = .039, η_p_^2^ = .099) revealed main effects of emotion for electrode pairs O9 and PO9, and O10 and PO10 (F(2, 48) = 10.002, *P* < .001, η_p_^2^ = .294) and F(2, 48) = 9.013, *P* < .001, η_p_^2^ = .273), with afraid faces eliciting less positive amplitudes than neutral (t(24) = −3.92, *P* = .002) and happy faces (t(24) = −2.7, *P* = .038), and more negative afraid-neutral emotion indices (t(24) = −2.69, *P* = .013 and t (24) = −3.58, *P* = .001).

### Main effects of emotion and task on Visual Evoked Potentials (VEPs) across groups

For both groups, main effects of emotion were observed for all VEP components confirming the effectiveness of the visual emotion manipulation. P120 showed a main effect of emotion (F(2, 98) = 10.481, *P* < .001, η_p_^2^ = .176), with less positive amplitudes for afraid compared to neutral (t(50) = −3.75, *P* = 0.001) and happy faces (t(50) = −2.92, *P* = .016), and a significant emotion index difference (t(50) = −2.862, *P* = .006). An emotion x hemisphere interaction (F(2, 98) = 7.994, *P* = .001, η_p_^2^ = .140) revealed main effects of emotion in both hemispheres (right: F(2,100) = 17.303, *P* < .001, η_p_^2^ = .257; left: F(2,100) = 6.881, *P* = .002, η_p_^2^ = .121), with less positive amplitudes for afraid compared to neutral and happy faces.

N170 showed a main effect of emotion (F(1.79, 89.68) = 24.150, *P* < .001, η_p_^2^ = .326), with less positive amplitudes for afraid compared to neutral (t(50) = −5.90, *P* < .001) and happy (t(50) = −3.16, *P* < .001) faces, and more positive amplitudes for neutral compared to happy faces (t(50) = 4.33, *P* < .001), and a significant emotion index difference (t(50) = −3.156, *P* = .003). N170 also showed a task x emotion interaction. Followed up by task, the emotion discrimination task showed a main effect of emotion (F(2,100) = 5.279, *P* = .007, η_p_^2^ = .096) with neutral being significantly different than afraid (t(50) = 2.93, *P* = .016). The sex discrimination task showed a main effect of emotion (F(2,100) = 23.540, *P* < .001, η_p_^2^ = .320) with significant differences between neutral and afraid (t(50) = 5.83, *P* < .001), neutral and happy (t(50) = 4.57, *P* < .001), afraid and happy (t(50) = −2.91, *P* = .016) and the emotion index difference (t(50) = −2.91, *P* = .005).

P200 showed a main effect of emotion (F(2, 100) = 11.775, *P* < .001, η_p_^2^ = .191), with less positive amplitudes for afraid compared to neutral (t(50) = −4.11, *P* < .001) and happy faces (t(50) = −4.25, *P* < .001), and a significant emotion index difference (t(50) = −4.223, *P* < .001).

#### Group differences on Somatosensory Evoked Potentials (SEPs after subtracting activity from visual only conditions, VEP-free SEP)

Repeated measures ANOVAs on VEP-free SEP mean amplitudes included within-subject factors of task (emotion, sex), emotion (neutral, afraid, happy), hemisphere (left, right), region (anterior, central, posterior), and site (dorsal, dorsolateral, lateral), and between-subject factor of group (dancers, non-dancers). Key findings for SEPs revealed expertise-related modulations of somatosensory emotion processing, with distinct patterns emerging for dancers and non-dancers across different SEP components. “Region” refers to the anterior-central-posterior grouping of somatosensory electrodes horizontally and 'site' refers to the dorsal-dorsolateral-lateral grouping vertically.

#### P50 (38–68 ms): Early expertise effect on emotion in central somatosensory regions

A significant group x emotion x hemisphere x region interaction (F(3.17, 155.53) = 3.321, *P* = .019, η_p_^2^ = .063) indicated that the pattern of emotion processing across hemispheres and regions differed between groups at this early somatosensory stage. Breaking down the interaction by group, dancers showed an emotion x hemisphere x region interaction (F(2.88, 72.03) = 4.261, *P* = .009, η_p_^2^ = .146). Follow-up analyses revealed main effects of emotion in central regions of both hemispheres (right: F (2, 50) = 3.286, *P* = .046, η_p_^2^ = .116 with non-significant post-hoc comparisons, neural vs afraid t(25) = 0.03, *P* > .9, neutral vs happy t(25) = 2.01, *P* = .162 and afraid vs happy t(25) = 2.47, *P* = .062; left: F(2,50) = 4.045, *P* = .024, η_p_^2^ = .139). In the left central SCx, neutral faces elicited negative amplitudes, while afraid faces elicited positive amplitudes (t(25) = −3.45, *P* = .006), suggesting early somatosensory differentiation of emotions in dancers (Fig. 3). Control participants did not show any significant emotion effects or interactions at the P50 component.

**Figure 3:**
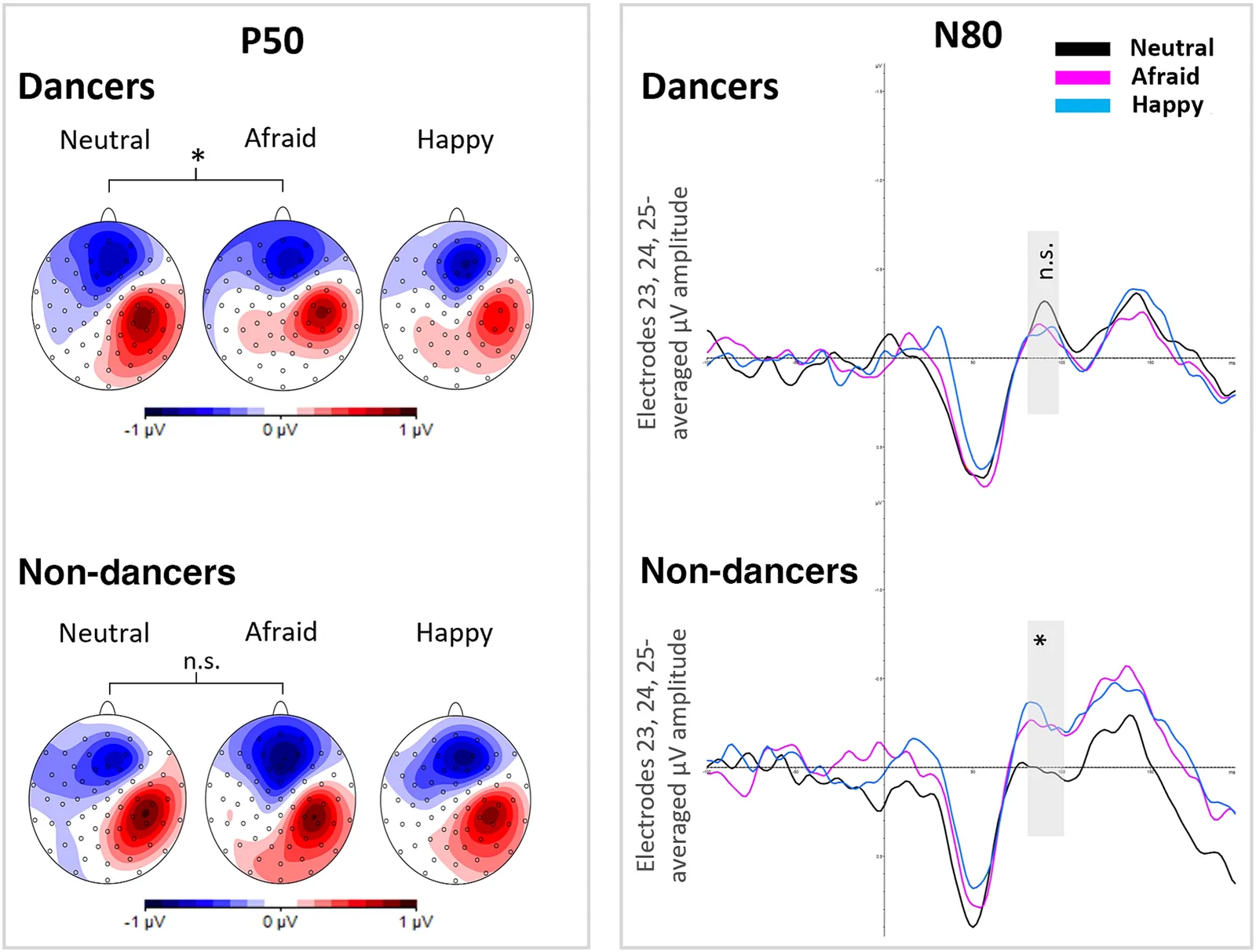
The emotion effects on each group for the somatosensory evoked potentials after subtracting the visual carry- over effects at P50 and N80. *Note*: On the left the topographical maps of the electrophysiological activity in 60 electrodes show the emotion effect found only on dancers at P50 with significant differences between afraid and neutral expressions. This effect was not found on non-dancers. The figures on the left show the SEP waves for each group on electrodes 23, 24 and 25 (corresponding to F06, C6 and CP6 on right lateral SCx). Only control participants had a significant emotion effect at N80 on these electrodes. This effect was not found on dancers.

#### N80 (69–99 ms): Expertise modulation of task and emotion

At the N80 SEP component, dancers showed task and emotion interactions, with higher amplitudes for the emotion task in the left hemisphere and task-related spatial variations in the right hemisphere. Dancers also showed an overall main effect of emotion with distinct responses to neutral and afraid faces. In contrast, control participants showed emotion effects primarily in the left hemisphere and at lateral sites, with stronger positivity for afraid faces in the left hemisphere, and differential responses to neutral and happy faces at right lateral sites (Figs 3 and 4).

**Figure 4:**
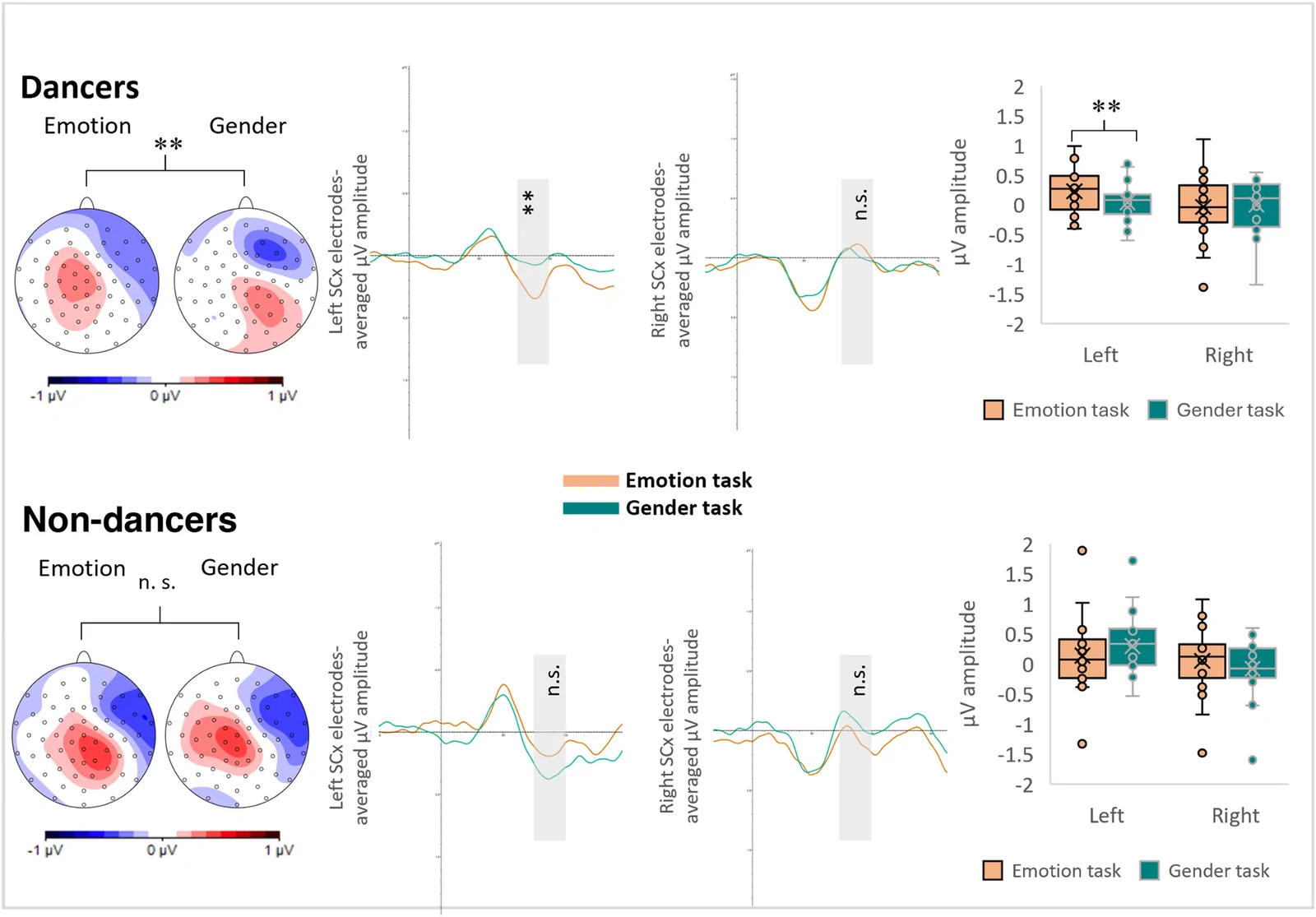
The task effect for the somatosensory evoked potentials after subtracting the visual carry- over effects at N80. *Note*: On the left the topographical maps of the electrophysiological activity in 60 electrodes show the significant task effect found only on dancers only and not on non-dancers. The central figures show the SEP waves across the left and the right SCx electrodes for each group. The boxplots on the right show the amplitude distribution and individual data points for the averaged activity on emotion and sex task on right and left SCx for each group. Dancers had a significant emotion effect on the left hemisphere with a positivity peak for emotion task amplitude. On the right hemisphere dancers had a significant task x region interaction and a task x site interaction. Non-dancers did not show any significant effects on task.

##### Expertise effects on task

N80 revealed several significant interactions involving group. The group x task x hemisphere x region interaction was significant (F(2, 94) = 5.747, *P* = .004, η_p_^2^ = .105). Breaking the interaction down by group, dancers showed a task x hemisphere x region interaction (F(2, 50) = 8.454, *P* < .001, η_p_^2^ = .253). In the right hemisphere, a task x region interaction (F (2, 50) = 5.794, *P* = .005, η_p_^2^ = .188) showed that the sex discrimination task elicited more negative amplitudes in the anterior region, similar amplitudes in the central region, and more positive amplitudes in the posterior region compared to the emotion task (non-significant post-hoc comparisons). In the left hemisphere, a main effect of task (F(1, 25) = 4.830, *P* = .037, η_p_^2^ = .162) indicated more positive amplitudes for the emotion discrimination task than the sex discrimination task (Figs. 3 and 4). Follow-up analysis in the control group showed no significant effects related to task at N80.

The group x task x hemisphere x site interaction was also significant (F(1.77, 86.75) = 5.412, *P* = .008, η_p_^2^ = .099). Breaking down the interaction by group, dancers showed a task x hemisphere x site interaction (F(2, 50) = 3.606, *P* = .034, η_p_^2^ = .126). The right hemisphere showed a task x site interaction (F(1.34, 33.39) = 5.071, *P* = .022, η_p_^2^ = .169), with similar task amplitudes at dorsal and dorsolateral sites and more negative amplitudes for the emotion task at lateral sites. The left hemisphere showed the main effect of task described earlier. The follow up analysis for control participants did not show any significant task-related effects at N80.

##### Expertise effects on emotion

The group x emotion x hemisphere x site interaction was significant (F(3.07, 150.51) = 4.736, *P* = .003, η_p_^2^ = .088). Following up the interaction by group, dancers exhibited a main effect of emotion (F(2, 50) = 4.834, *P* = .012, η_p_^2^ = .162), with negative amplitudes for neutral and positive amplitudes for afraid faces (t(25) = −3.10, *P* = .014), and a more positive afraid-neutral emotion index than happy-neutral index (t(25) = 2.435, *P* = .022).

Control participants showed an emotion x hemisphere interaction (F(2, 48) = 3.404, *P* = .044, η_p_^2^ = .124) and an emotion x hemisphere x site interaction (F(2.69, 62.2) = 4.084, *P* = .014, η_p_^2^ = .145). Main effects of emotion were found in the left hemisphere (F(2, 48) = 4.320, *P* = .019, η_p_^2^ = .153), and at lateral sites of both hemispheres (right: F(2, 48) = 3.515, *P* = .038, η_p_^2^ = .128; left: F(2, 48) = 3.877, *P* = .027, η_p_^2^ = .139). In the left hemisphere, afraid faces elicited more positive amplitudes than neutral (t(24) = −2.73, *P* = .036). At the right lateral site, neutral faces elicited positive and happy faces negative amplitudes (t(25) = 2.59, *P* = .048).

#### P100 (99–119 ms): Expertise effects on task and emotion

At the P100 SEP component, dancers showed interactions involving task and emotion together, with emotion effects in left central and posterior regions. Control participants showed task and emotion interacting independently with hemisphere, showing effects at left lateral sites.

The group x emotion x task x hemisphere x region interaction was significant at P100 (F(2.96, 144.92) = 3.843, *P* = .011, η_p_^2^ = .073). Breaking down by group, dancers showed an emotion x task x hemisphere x region interaction (F(2.66, 66.53) = 4.497, *P* = .008, η_p_^2^ = .152). In the left hemisphere, there was a task x region x emotion interaction (F(4, 100) = 4.632, *P* = .002, η_p_^2^ = .156) that we followed by region and by emotion. Focusing on region, we found main effects of emotion in the central (F (2, 50) = 3.438, *P* = .040, η_p_^2^ = .121, non-significant post-hoc) and posterior regions (F(2, 50) = 3.850, *P* = .028, η_p_^2^ = .133), with positive amplitudes for afraid and negative for happy faces (t(50) = 2.65, *P* = .041). Focusing on emotion, we found a task x region interaction only for the happy faces (F(1.15, 28.873) = 9.064, *P* = .004, η_p_^2^ = .266) and a main effect of task on the left anterior SCx (F1, 25) = 5.729, *P* = .025, η_p_^2^ = .166).

Control participants showed a task x hemisphere interaction (F(1,24) = 4.685, *P* = .041, η_p_^2^ = .163), with more negative emotion task amplitudes in the right hemisphere and more positive in the left.

The group x emotion x hemisphere x site interaction was also significant (F(3.02, 147.85) = 3.259, *P* = .023, η_p_^2^ = .083). Breaking the interaction down by group, control participants showed an emotion x hemisphere x site interaction (F(2,91, 69.77) = 3.741, *P* = .016, η_p_^2^ = .135), with a main effect of emotion at the left lateral site (F(2, 48) = 3.324, *P* = .044, η_p_^2^ = .122, non-significant post-hoc). We found no significant interaction follow-ups for dancers.

#### N140 (119–145 ms): Expertise effect on task

The group x emotion x task x region interaction was significant (F(2.28, 111.83) = 3.466, *P* = .029, η_p_^2^ = .066). Dancers showed an emotion x task x region interaction (F(2.27, 56.72) = 4.913, *P* = .008, η_p_^2^ = .164), and further analysis revealed a task x region interaction specifically for happy faces (F(1.24, 30.93) = 4.860, *P* = .028, η_p_^2^ = .162) and a main effect of region for sex discrimination task only (F(1.44, 36.11) = 4.298, *P* = .032, η_p_^2^ = .147).

The group x emotion x task x site interaction was also significant (F(2.76, 135.32) = 5.287, *P* = .002, η_p_^2^ = .097). Breaking down the interaction by group, non-dancers showed an emotion x task x site interaction (F(2.75, 65.87) = 3.972, *P* = .014, η_p_^2^ = .142), and within the sex discrimination task, main effects of site were found for neutral (F(1.44, 34.66) = 5.776, *P* = .013, η_p_^2^ = .194) and happy faces (F(1.2, 28.85) = 7.147, *P* = .009, η_p_^2^ = .229). Dancers did not show significant effects for emotion or task on the follow up analysis.

### Main effects of emotion and task on Somatosensory Evoked Potentials (SEPs) across groups

Across groups, main effects of emotion and interactions with hemisphere and task were observed for SEPs. P50 showed a task x emotion x region interaction (F2.17, 106.6) = 3.111, *P* = .044, η_p_^2^ = .06). Follow-up analysis showed on neutral faces only a main effect of task (F(1,50) = 6.918, *P* = .011, η_p_^2^ = .122) and a task x region interaction (F (1.19, 59.31) = 10.413, *P* = .001, η_p_^2^ = .172) followed by a main effect of task on anterior SCx only (F(1,50) = 6.330, *P* = .015, η_p_^2^ = .112).

N80 showed a main effect of emotion (F(2, 100) = 5.449, *P* = .006, η_p_^2^ = .098), with more positive amplitudes for neutral compared to afraid faces (t(50) = −3.57, *P* = .002), and a more positive afraid-neutral emotion index than happy-neutral index (t(50) = 2.006, *P* = .05). An emotion x hemisphere interaction (F(2, 98) = 3.878, *P* = .024, η_p_^2^ = .073) revealed a main effect of emotion in the left hemisphere only (F(2, 100) = 10.479, *P* < .001, η_p_^2^ = .173), with less positive amplitudes for neutral compared to afraid and happy faces.

P100 showed again the task x emotion x region interaction (F(2,325, 113. 92) = 3.842, *P* = .019, η_p_^2^ = .073). Follow-up analysis showed on afraid faces only a main effect of task (F(1, 50) = 4.626, *P* = .036, η_p_^2^ = .085) and a task x region interaction (F(1.69, 84.313) = 4.580, *P* = .018, η_p_^2^ = .084) followed by a main effect of task on anterior SCx (F(1,50) = 5.952, *P* = 0.018, η_p_^2^ = 0.106) and central SCx (F(1,50) = 5.657, *P* = .021, η_p_^2^ = .102). P100 also showed an emotion x hemisphere interaction (F(2, 98) = 3.287, *P* = .042, η_p_^2^ = .063), with a main effect of emotion in the left hemisphere (F(2, 100) = 5.209, *P* = .007, η_p_^2^ = .094), showing negative amplitudes for neutral and positive for afraid faces (t(50) = −3.27, *P* = .006), and a more positive afraid-neutral emotion index than happy-neutral emotion index (t(50) = 2.247, *P* = .029).

## Discussion

This study investigated the visual and somatosensory processing of facial emotion expressions in professional ballet dancers, capitalizing on their unique artistic and sensorimotor expertise. The central hypothesis, that dance expertise modulates processing of facial emotions, is supported by our ERP findings. Group differences emerged across early ERP components in both visual and somatosensory modalities, indicating that dance expertise shapes fundamental aspects of emotion perception.

### Visual emotion processing: Expertise and holistic emotion representation

Our VEP findings reveal that dance expertise modulates visual emotion processing, particularly at the N170 and P200 components. Dancers exhibited a distinct pattern, showing less negative N170 amplitudes for neutral faces compared to emotional faces (afraid and happy), suggesting an early categorical distinction between neutral and emotional expressions related to sensitivity to emotional faces. This categorical pattern is consistent with literature on N170 reflecting holistic structural encoding of emotionally salient stimuli (Eimer and Holmes 2002, Williams et al. 2004, 2006). Furthermore, at P200, dancers differentiated between negative (afraid) and positive (happy) emotions consistent with evidence that later VEP components reflect more elaborated processing of facial affect, including valence-based differentiation by N170. The increased negativity for neutral faces in dancers at N170 suggests differences in sensitivity or attention allocation to emotionally neutral stimuli. These findings align with the emerging literature on artistic expertise and face processing (Seabra et al. 2022), extending it to the domain of performing arts and emotion perception.

Non-dancers showed a more graded emotion sensitivity at N170, with distinct responses to neutral, afraid, and happy faces, and differentiated between afraid and neutral/happy faces at P200. Non-dancers also exhibited more lateralized emotion effects, particularly in the right hemisphere, while dancers showed more generalized emotion effects across hemispheres. Non-dancers' right-lateralized N170 pattern is consistent with the well-established right-hemisphere dominance for configural face processing and facial emotion recognition, and their differentiation between all emotion categories at N170 reflects the amplitude modulation by emotional expression documented in the broader ERP literature (Williams et al. 2004, 2006; Conty et al. 2012).

The overall main effects of emotion across VEP components (P120, N170, P200) confirm the effectiveness of our visual emotion manipulation and are consistent with established literature on facial emotion processing (Hinojosa et al. 2015). These VEP patterns suggest that dance expertise modulates the categorical organization of emotional salience at early visual stages and the valence-based differentiation between emotional categories at later stages. The two group patterns reflect qualitatively distinct styles of emotion discrimination: non-dancers showing a graded, right-lateralized response consistent with the typical N170 emotion literature, and dancers showing a more categorical and bilaterally distributed pattern.

#### Somatosensory emotion processing: Expertise and embodied emotion

The SEP findings provide novel insights into the embodied nature of emotion processing and its modulation by dance expertise both at early components (P50, N80) reflecting mainly primary somatosensory processes and middle latencies (P100, N140) reflecting secondary and higher order somatosensory processes (Vibell et al. 2023). At the early P50 component, dancers uniquely showed emotion effects in central somatosensory regions, showing differential responses to neutral and afraid faces but not to neutral and happy faces, indicating that this effect is specific to afraid expressions and not a general response to emotionally salient stimuli. This modulation, occurring at 38 to 68 ms post-tactile onset (approximately 143 to 173 ms after face onset), reflects early sensory-level somatosensory engagement during emotion perception. At N80, dancers displayed complex interactions involving both task and emotion, and the afraid-neutral emotion index was significantly larger than the happy-neutral index, indicating that the two emotional categories were not processed equivalently and that afraid expressions were the primary driver of early somatosensory differentiation. In contrast, non-dancers showed emotion effects primarily at later stages (N80 and P100) and with a more localized pattern, particularly at lateral sites, suggesting a less generalized somatosensory engagement in emotion processing compared to dancers. The main effect of emotion observed in dancers at N80, with distinct responses to neutral and afraid faces, aligns with the growing body of evidence supporting embodied emotion theories (Sel et al. 2014, 2020; Arslanova et al. 2023), and extends this to the context of artistic expertise. These studies have demonstrated emotion-specific somatosensory differentiation between emotional categories including fearful vs. happy contrasts using the same paradigm, supporting the interpretation that the present effects reflect differential processing across emotional categories rather than an undifferentiated response to emotional salience. The task-dependent group differences observed are also consistent with previous research highlighting the role of task demands in modulating somatosensory emotion processing (Fanghella et al. 2022). The distinct patterns of somatosensory activity in dancers and non-dancers across SEP components provide compelling evidence for the modulation of somatosensory engagement during emotion processing by dance expertise, consistent with embodied accounts of emotion perception (Pourtois et al. 2004; Pitcher et al. 2008; Sel et al. 2014; Fanghella et al. 2022, 2026).

#### Expertise effects, interoception, and the embodied perception of emotion

This study provides the first empirical evidence that dance expertise modulates early activations in both visual and somatosensory areas during the observation of facial emotion expressions. Dancers appear to show a broader, more distributed pattern of processing of emotion, as indicated by the less lateralized emotion effects in VEPs, and the early and task-modulated somatosensory engagement in SEPs despite the unilateral tactile stimulation on the left index finger. The finding that dancers showed emotion effects irrespective of the task, unlike the more task-specific emotion effects in non-dancers, further supports this interpretation. Both groups, however, showed distinct processing of afraid expressions, reinforcing the established dominance of fear in emotion processing (Batty and Taylor 2003; Pegna et al. 2008) possibly with evolutionary links. Anecdotally, fear is an important tool in dance training, for example as dancers learn how to feel comfortable with exposure or performance in front of audience.

Our results provide a pivot point to the classic literature describing specific effects of dance training in body and movement perception (Calvo-Merino et al. 2005 and 2006; Cross et al. 2006; Orgs et al. 2008; Orlandi et al. 2017; Orlandi and Proverbio 2019). While previous work has focused on describing visual (Calvo-Merino et al. 2010) or emotional sensitivity to dance stimuli (Christensen et al. 2016a), here we tested the expertise effect beyond the domain specific dance movement. The visual and somatosensory modulation to the face emotion processing suggest this expertise effect can be generalized to other non-dance stimuli.

In addition, the significantly higher MAIA scores in dancers, particularly in subscales related to body awareness and emotion regulation, further extend the link between dancer’s enhanced interoceptive abilities (Christensen et al. 2017b) and expertise-related modulations in emotion processing (Christensen et al. 2014), with a generalized effect on emotion processing that includes facial expressions.

Finally, the early differential effects between dancers and non-dancers in the somatosensory activity (P50, N80) controlled for visual effects, suggest that the embodiment of facial expressions previously described (Sel et al. 2014) is modulated by the sensorimotor expertise of the dancers. This result complements other embodiment modulations in somatosensory cortices described in other group comparisons, namely individuals with ASD with neurotypical individuals (Fanghella et al. 2022, 2026).

#### Limitations and future directions

A key strength of this study is the novel investigation of facial emotion processing in professional ballet dancers, a unique population with combined artistic and sensorimotor expertise. With the use of both visual and somatosensory ERP measures, combined with a rigorous ERP subtraction method to isolate VEP-free SEPs, we provide a comprehensive examination of emotion processing. The inclusion of psychometric measures, particularly the MAIA, adds valuable insights into the role of interoception and personality traits. However, the study also has limitations. The study followed sample sizes reported in the literature. Future studies should include power analyses a priori and be pre-registered. Further research is needed to precisely localize the sources of the observed differential somatosensory activity, potentially using neuroimaging methods with higher spatial resolution, such as MEG or fMRI, in conjunction with EEG for improved spatiotemporal resolution. Tactile stimulation in a different area (for example right index finger) or multiple areas (Sel et al. 2020) could shed light into the localization differences between dancers and non-dancers. Future studies could also explore the incorporation of trial-based behavioral measures of emotion recognition and discrimination to observe if the neural differences are accompanied by measurable behavioral differences.

Finally, in line with previous work exploring the developmental trajectory of these experience-related effects in interoception (Christensen et al. 2017b) and sensorimotor resonance during action perception (Cross et al. 2006; Kirsch et al. 2015), new studies could explore how somatosensory engagement during emotion processing evolves across stages of dance training, from advanced trainees to professional performers. Similariy, further exploring the relationship between embodied emotion and interoceptive sensitivity as trainable skills, and how these findings can extend to other forms of artistic and motor expertise, would consolidate a new connection between artistic training and body related processes.

## Conclusion

In conclusion, this study provides novel empirical evidence that dance expertise modulates early visual and somatosensory activations during the observation of facial emotion expressions. These findings offer new insights into the nature of expertise, particularly artistic and motor expertise, demonstrating its influence on both the visual and embodied perception of emotions. Our results align with and extend previous literature on dance expertise and embodied cognition, highlighting the profound impact of specialized training on fundamental aspects of human emotion processing and perception, with broader implications for understanding the interplay between art, the body, and the brain.

## Data Availability

The EEG files in a non-identifiable form are available at: 10.25383/city.31302025
